# Correlation between Isotherms and Isodoses in Breast Cancer Radiotherapy—First Study

**DOI:** 10.3390/ijerph18020619

**Published:** 2021-01-13

**Authors:** Dominika Plaza, Agnieszka Baic, Barbara Lange, Agata Stanek, Krzysztof Ślosarek, Anna Kowalczyk, Armand Cholewka

**Affiliations:** 1 Radiotherapy Planning Department, Maria Skłodowska—Curie National Research Institute of Oncology Gliwice Branch, Wybrzeże Armii Krajowej Street 15, 44-102 Gliwice, Poland; dominikaplaza1@gmail.com (D.P.); barbara.lange@io.gliwice.pl (B.L.); krzysztof.slosarek@io.gliwice.pl (K.Ś.); 2 A. Chełkowski Institute of Physics, Department of Medical Physics, University of Silesia, 75 Pułku Piechoty 1A, 41-500 Chorzów, Poland; armand.cholewka@gmail.com; 3 Department and Clinic of Internal Diseases, Angiology and Physical Medicine in Bytom, School of Medicine with the Division of Dentistry in Zabrze, Medical University of Silesia, Batorego Street 15, 41-902 Bytom, Poland; astanek@tlen.pl; 4 Department of Physiotherapy, School of Health Sciences, Katowice Medical University of Silesia in Katowice, Poniatowskiego Street 15, 40-055 Katowice, Poland; akowalczyk@sum.edu.pl

**Keywords:** infrared thermography, radiotherapy, isodoses, isotherms

## Abstract

The study is focused on correlation of isotherms derived from thermal images with an isodoses describing treatment plan for patients with breast cancer treated by radiotherapy. The irradiated area covered the part of the body after mastectomy. The study included patients diagnosed with breast cancer who were qualified for radiotherapy treatment. All patients were monitored during each treatment week during the entire radiotherapy process. The measurements were made under strictly defined conditions. In the treatment planning system (TPS), the specific plan was created for each patient. Spatial dose distribution in the patient’s body was obtained and presented by the isodoses (lines connecting points with the same dose values). The following areas from the treatment planning system were plotted on the thermograms: target (tumor area) and isodose: 45 Gy, 40 Gy, 30 Gy, 20 Gy and 10 Gy. The obtained results indicated a high correlation between magnitude of the dose represented as the isodose and the temperature of the treated skin. Moreover, preliminary analysis showed a repeatable increase of the mean temperature in the irradiated area during the treatment.

## 1. Introduction

Nowadays breast diseases are one of the most serious and important clinical health problems. Moreover, as far as breast cancer is concerned, incidence and mortality are rising in every developed country, including Poland, at an alarming rate. This is evidenced by the morbidity indicator, which is 96 per 100,000 inhabitants of Western Europe. On the other hand, in developing countries (such as Central Africa and East Asia), the incidence rate is 27 per 100,000 inhabitants [1]. A different pattern of procreation, insufficient physical activity, obesity and genetic predisposition (mutation of the BRCA1 and BRCA2 genes) are the main risk factors that have a documented impact on morbidity. These effects require the introduction into clinical practice of an easy-to-use and non-invasive imaging method that will enable early diagnosis or evaluation of the therapeutic effects of breast diseases [2,3,4,5,6].

Fortunately, modern medicine offers a wide range of specialized diagnostic methods that can be adapted to the shape, size, location and stage of breast cancer, and can also confirm or exclude the presence of pathological neoplastic tissue. A mammography is currently one of those methods and one of the most effective methods of early diagnosis—it uses a series of X-ray images absorbed by breast tissue. The second method is ultrasonography (USG), which is dedicated to women aged 20–40. Ultrasonography (USG) is the useful complementary technique to mammography and medical history or interview. Biopsy (thin-needle biopsy, core-needle biopsy) is one of the techniques used for microscopic analysis and identification of breast tissues. Moreover, it can be used in addition to surgery to remove benign, small tumors in the diagnostic process. Because of the fact that ionizing radiation is harmful to a patient’s health and can affect the human body, it became clear and obvious that contemporary, modern medicine needs new diagnostic methods that would not be associated with the risk of side effects but instead would provide us with the same (or even a higher) accuracy for diagnostic results, repeatability of tests and patient comfort. All these aspects explain why thermography is increasingly used in the diagnostic and therapeutic process. The idea of measuring breast temperature to detect cancerous tissue is no different from other diagnostic methods and techniques that rely on (and use) thermography. The images obtained by thermography should refer to the anatomical shape of the breast—therefore, the explorer has to obtain several thermograms (of the tissue) in different projections. Thermal asymmetry between the mammary glands may indicate the presence of cancerous tissue (if there is no other possible factor that could alter the surface temperature of the skin—e.g., abrasions). Initially, thermal imaging was not a popular method in the scientific community. This changed in the 1980s thanks to technological advances in infrared sensing and greater/wider access to smarter computers. These changes made it possible to improve the quality of the thermography method by increasing its diagnostic value, its accuracy and reliability of the results [7].

In thermal (imaging) diagnostics of breast glands, it is very important to learn about the process of carcinogenesis or breast physiology, as well as the basics of endocrinology and physics that describe processes such as creating a temperature map of a given surface part of the body. Thermal imaging is not the easiest method to interpret. It is important to know and be aware that the severity of a tumor depends on the degree of differentiation of the cancer cells. Neoplastic cell atypia is most often associated with the advanced stage of cancer and its acceleration/dynamic progress. It is well known that the stage of cancer should be determined prior to treatment, and the assessment of cancer stage should take into account the size of the tumor, the presence of cancer cells in the axillary lymph nodes and the frequency of metastasis to distant cell sites. As for the use of thermography, which can be used during the process of breast disease, it is worth stating that this technique is very helpful and can give us information about deviations, changes and pathologies in breast physiology, including about angiogenesis or changes in metabolic activity (but not about the anatomical structure of the breast glands). These two pieces of information are much more important and relevant to the risk of cancer, as physiological changes are observed and revealed much earlier than changes in the structure of the breast.

Thermovision is a non-invasive and contactless imaging method. It is fully safe due to patients not being exposed to ionizing radiation. Thermal imaging brings some functioning information, indirectly connected with metabolism, so it may be used in screening tests or as a complement to existing and currently used diagnostic methods, like mammography or ultrasonography (USG) [8,9,10,11,12].

A growing and accelerating tumor needs nutrients that can be transferred to it through the bloodstream. It is preceded by neoangiogenesis, which enables the transport and distribution of blood and blood components to the tumor [13]. Angiogenesis is a very complex process involving various mediators and enzymes. Vascular endothelial growth factor (VEGF) is one of the most important factors in this process and is synthesized by vascular/vascular cells. VEGF stimulates the development of a network of blood vessels and increases their permeability. Increased levels of this factor have been reported in many different types of cancer, including breast cancer. VEGF concentrations are directly proportional to the size and density of the blood vessel network in the tumor area and are associated with low or high grade/tumor grade. Nitric oxide (NO) is another important factor influencing the process of blood vessel formation [14]. It does NOT occur naturally in the human body. It is excreted by endothelial cells and this process activates angiogenesis. It has been fully proven that the concentration of NO in cancer cells is much higher than in healthy cells of the body. This concentration is also proportional to the temperature, so its increase in the superficial layer of the skin is clearly visible in thermal images [15]. The formation of the vascular network, the transport of nutrients through these new vessels and the stimulation of the smooth muscles of the blood vessels affect the metabolism of the human body, and thus the heat map of the body. This map is visible on the surface of the body and involves skin vascularization (sufficient blood supply to the skin provided by the small blood vessels and skin capillaries). Changes in temperature distribution in a healthy body are usually symmetrical—meaning they are almost the same in both mammary glands. Thermal asymmetry can be caused by tumors close to the surface (and changes in their vascularization), but also by tumors deeper that are known to be absorbed into the main blood vessel. Thermovision made it possible to detect a small neoplastic tissue much earlier than other diagnostic methods, because the analysis and examination of thermograms allows us to show even the smallest differences and changes in the vascularization of the mammary glands [16,17,18,19]. The effects are especially pronounced when considering the location of the tumor near the skin surface. The deeper the tumor is, the more difficult it is to see it on thermal images. Higher levels of nitric oxide (NO) dilate the blood vessels—the temperature of the breast tissue is higher, and this is a data set that can be recorded using infrared thermography (thermal imaging).

Easier access to digital infrared imaging could make this method one of the most reliable and widely used screening techniques that can be used to assess and detect diffuse breast tumor and further characterize focal breast disease [3,20,21,22,23,24].

The frequency of diagnosis of breast cancer, its early detection and the fastest possible treatment are often crucial for patients. There are several treatment options for this type of cancer, ranging from forms of systemic treatment such as hormone therapy and chemotherapy to forms of local treatment such as radiation therapy. In most cases, combination therapy is used. Radiotherapy is a local treatment that uses ionizing radiation to kill cancer cells. The following types of radiotherapy are distinguished: teleradiotherapy (EBRT), where treatment is planned from a source outside the body at a distance from irradiated tissues using a linear accelerator; and brachytherapy (BRT), where the source used for treatment is in direct contact with the tumor. The amount of radiation that is delivered to the patient’s body is called the dose. The unit of radiation dose is gray (Gy) or centigray (cGy; 1 Gy = 100 cGy). The radiation dose of 1 Gy means that the energy of 1 J has been absorbed on the weight of 1 kg of tissue. Most often, the entire dose is divided into smaller portions, called fractions. Different dosages are used for different types of cancer and at different stages of treatment. Therefore, the duration of radiotherapy depends on the dose and the number of fractions. The dose fraction is typically administered once a day or every 2 days. In some cases, it is necessary to administer 2 fractions in one day. The patient is informed about the dose fractionation method and the associated treatment duration before starting therapy. Radiation therapy (irradiation) is a special type of procedure that precisely targets the affected area with the intention of healing (radical radiotherapy) or slowing down the course of the disease and/or relieving its symptoms, such as pain, bleeding, shortness of breath, difficulty swallowing and many other symptoms (radiation therapy, also known as palliative therapy). Radical radiation therapy is usually performed for 4 to 7 weeks.

In the presented work, the treatment uses linear accelerators emitting high-energy photons and electrons. Therefore, the volume of irradiated healthy tissue was limited to a small margin around the tumor. Consequently, the dose to the tumor may be increased to achieve a therapeutic effect. Irradiation planning is based on a computed tomography (CT) examination. In CT layers in the treatment planning system (TPS), the radiotherapist determines the tumor boundaries, obtaining the area of the solid tumor (GTV—Gross tumor volume). Tumor margins around the microscopic tissue are then marked to obtain the clinical target area (CTV, clinical target volume). The margin is added to the GTV and CTV to achieve the Planned Target Volume (PTV). A commonly used technique for treating breast cancer is computed tomography-planned conformal radiotherapy, which uses tangent and cassette fields (if necessary). However, dynamic techniques such as intensity modulated radiotherapy (IMRT) or volumetric modulated arc therapy (VMAT) are now used much more frequently. The use of dynamic techniques allows for precise adjustment of the spatial distribution of the radiation dose to the individual characteristics of the tumor, such as size, shape and location. During a single radiation therapy session, the medical accelerator enables the delivery of different doses of radiation to different target areas. Optimization of the treatment plan in both dynamic techniques allows for an even distribution of the dose in the irradiated area, reducing the dose in the organs critical for this location, i.e., the lung on the side of the tumor, the heart or head of the humerus [25]. For the success of radiotherapy and the comfort and quality of treatment for the patient, it is very important to ensure daily repeatability of the established irradiation conditions. Additional devices that immobilize the head, torso and limbs help in this task. Both the position and alignment of the patient’s body are controlled by laser centrators. This technology made it possible to accurately see the isodoses. However, a fast, safe and simple technique for radiotherapy effects evaluation is still needed. Such a technique may be thermal imaging which brings information about temperature distribution. It should be noted that absorbed energy during radiotherapy changes skin temperature distribution, which may be easily evaluated by using thermal imaging. Thermal imaging performed after radiotherapy can provide important information about temperature distribution and also do so indirectly about dynamics of metabolism changes. On the other hand, such information may lead to conclusions about the rebuilding of irradiated tissue, neoangiogenesis and the occurring of the inflammatory state and its range changing, thereby providing important information about the healing process and its dynamics.

That is why the main goal of this work was to compare and correlate the isodoses generated in the treatment planning system with isotherms obtained from thermal image, as well as to show the possibility of using thermal imaging to assess the effects of radiotherapy treatment.

## 2. Materials and Methods

Our study included 8 patients with diagnosed breast cancer after mastectomy who were qualified for ionizing radiation treatment. The age group of the respondents was 50–70 years (mean age 64.4 ± 5.94). The mean values of body weight and height of the participants were 72.4 ± 9.83 kg and 1.65 ± 0.6 m, respectively. Patients’ body mass index (BMI) ranged from 23.6 to 31, with a mean value of 26.64 ± 3.19 kg/m^2^.

All patients were controlled throughout the radiotherapy process. Before the study, the patients were informed about the method of conducting the experiment; they received the appropriate qualification questionnaire and the consent forms for examination. The measurements were made under strictly defined conditions (the patients remained without waist-length clothing for about 20 min) and were preceded by the acclimatization process at the temperature of the test room. Each of the examined patients had a thermal image taken with raised hands on the right and left side according to the guidelines [26,27,28,29,30,31].

Measurements were made with a specialized FLIR System E60 thermal imaging camera with a detector resolution of 320 × 240 pixels, the thermal sensitivity of which is 0.05 K.

The researchers maintained the temperature of the test room on a basic level of 22 °C ± 1 °C. The humidity in the room was maintained in the range of 40% to 45%.

The research aimed to observe the evolution of temperature parameters in each week of the treatment. Clinical data was collected to evaluate the impact of additional factors that were included in the questionnaire. These factors include age, BMI, medical history, pregnancy, lactation and genetic predisposition. In addition, at each stage of the therapy, patients were interviewed about the side effects of radiotherapy.

The entire research project was approved by the Bioethics Committee at the Oncology Center—Maria Skłodowska-Curie Institute in Warsaw on 6 October 2016, as confirmed by opinion No. 38/2016.

Patients were radiated by high-energy photons 6 MV with Varian’s TrueBeam linear accelerators.

**Figure 1:**
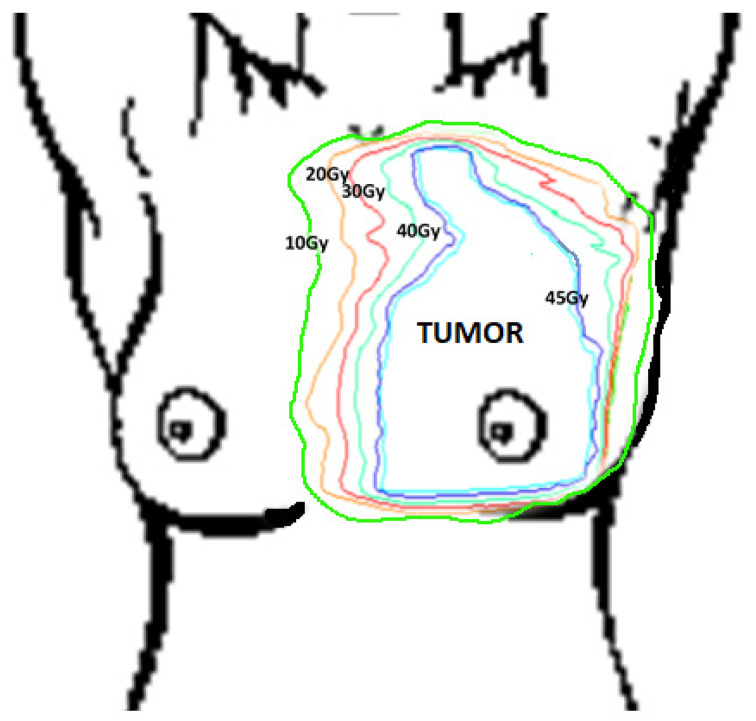
The scheme of drawing the isodoses on all thermograms with marked regions of interest (isodoses 10–45 Gy). Isodoses were obtained from the treatment planning system and were reflected on the thermal images.

The plan for each patient was prepared within the Treatment Planning System (TPS). As a result, a spatial distribution of the dose in the patient’s body was obtained and presented in the form of isodoses (lines connecting points with the same dose values). On each obtained thermogram, the following areas from the Treatment Planning System were marked: target (tumor area) and isodoses: 45 Gy, 40 Gy, 30 Gy, 20 Gy and 10 Gy. A diagram presenting method of drawing isodoses on thermograms is shown below in Figure 1.

Target size and isodose were determined according to a treatment plan prepared individually for each patient. A diagram of the method of drawing isodoses on thermograms is presented below in Figure 1.

## 3. Results and Discussion

Through the individual treatment plans, patients received 45 Gy in fractions of 2.25 Gy per day from Monday to Friday. In this way, the side effects of radiotherapy are reduced and the effectiveness of the treatment is improved. The results obtained for the treated patients are summarized in Table 1, which presents the temperature changes in the target area (PTV—Planned Target Volume) for all the studied patients during treatment and in the control group [25].

**Figure 2:**
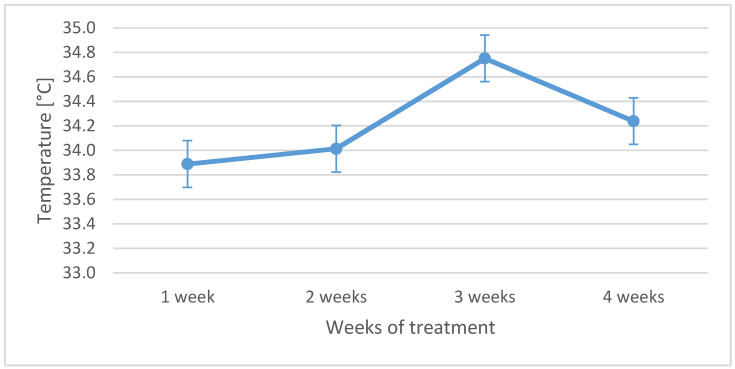
Average temperature changes for all examined patients during radiotherapy time.

The primary analysis was performed on average temperature obtained from thermal isotherms transferred from the thermal images of each patient. It should be underlined that the highest temperature was observed in the third week of the treatment for the entire group, as shown in the “temperature versus time” graph in Figure 2. In addition, the table shows that the control group has a significantly lower temperature in PTV than the treated group of patients.

**Figure 3:**
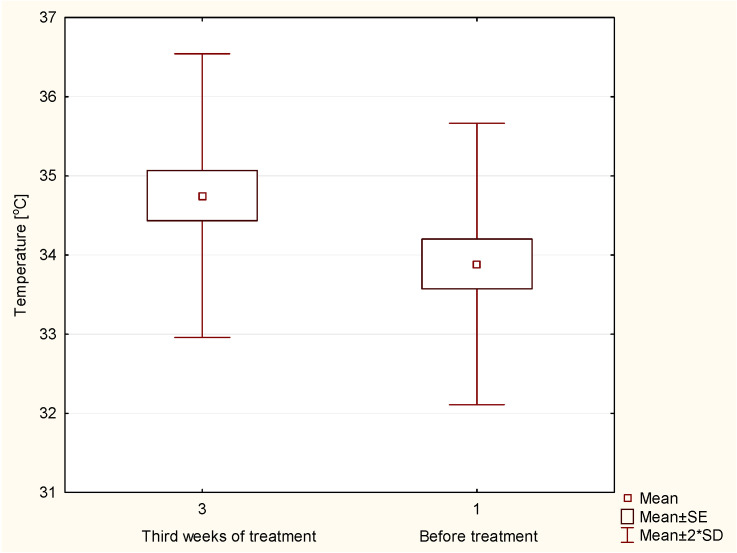
The temperature differences in target areas between the third week during the radiotherapy and before the treatment.

Figure 2 shows that the highest temperature increase was achieved in the third week of the treatment, which is consistent with the literature [32,33,34,35,36,37,38,39,40,41]. The temperature rise is seen at the Planned Target Volume (PTV) at which the patient dose was the highest. The increase in the average temperature in the PTV region was 0.78 °C. The differences in temperature in the target areas between the third week during radiotherapy and before treatment were statistically significant at *p* < 0.05, as shown in the graph boxes in Figure 3.

For a better insight into the problem, the areas of PTV and other isodoses from the treatment plan (40 Gy, 30 Gy, 20 Gy, 10 Gy) were analyzed and compared with thermal images. When analyzing subsequent isodoses, we can see that the temperature on the skin decreases with the dose decrease (Table 2). Outside the treatment area, healthy tissues receive a much lower dose of radiation, but this causes the body temperature to rise.

**Figure 4:**
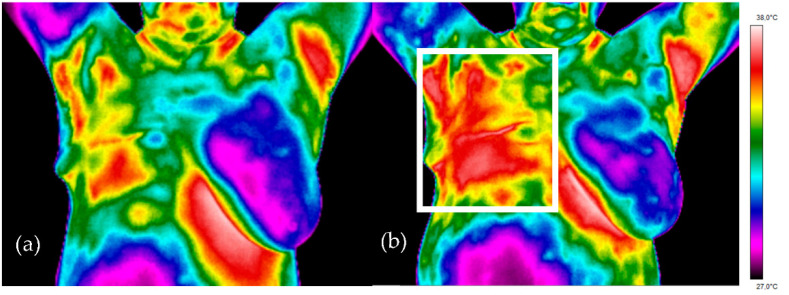
Patient after mastectomy. Treated area after right breast excision (**a**) thermogram before radiotherapy treatment. (**b**) thermogram after three weeks of radiotherapy treatment.

Figure 4 shows a simple projection of thermal images for representative breast cancer patients treated with radiotherapy prior to radiotherapy (left) and after three weeks of the treatment (right). The temperature range for each patient was the same and ranged from 27 °C to 38 °C. Thermal asymmetry between the mammary glands can be easily noticed in a representative patient, as well as in other patients in the study group, even before treatment with radiotherapy. One can clearly see the differences in the heat map between the thermal image obtained in the first and third weeks after treatment. Three weeks after the irradiation, the area is characterized by a much higher temperature (the red-color area in the white frame on the right breast in Figure 4b) compared to that observed before the treatment.

It should be noted that depending on the advancement of treatment, not only the breasts are treated but also the lymph nodes so the dose is accepted in wider area of the body. Thermal imaging performed in proper position of patients (with hands up) allows for obtaining temperature maps while also taking the lymph nodes into account and comparing the isotherms with radiobiological processes that take place in chosen areas—not only the breast. In the analyzed data, all results also included the area of the axillary lymph nodes. It should be mentioned that the thermal reaction may be different in the treated patients due to the use of various treatment techniques—modern irradiation techniques reduce the undesirable dose beyond the PTV area and save healthy tissue (the area where the reaction occurs should be smaller). It is not possible to only irradiate the tumor area. The high dose area always also includes healthy tissue in the immediate vicinity of the tumor. This causes radiation complications, which can be divided into early and late complications. The time of clinical onset of these lesions depends on the lifetime of differentiated mature cells.

The skin reaction is a fairly common side effect of radiotherapy and individual patients can react to the treatment in few different ways. The degree of skin reaction patients will experience primarily depends on the dose of radiation that they receive. The variation in expression of radiation skin reactions might be attributed not only to the known radiation factors but also to the personal and genetic factors that join differently for each individual patient. Patients with cancer which were treated by radiotherapy could experience many of factors that impair healing such as malnutrition, stress, anemia, anorexia-cachexia, some metabolic alterations, impaired mobility, old age, disturbances in blood circulation, anxiety and neurological disorders. These conditions have the potential to be detrimental to the repair of the skin epithelium. In addition, such patients are likely to have been hospitalized for surgery and may have had, or are having chemotherapy. The close relationship between the total dose, volume of treated tissue, fractionation and treatment time is fundamental to predicting the severity of radiation skin reactions. Normal tissue tolerances, which is the point at which erythema, dry or moist desquamation or necrosis occur, are usually reported in terms of the dose of radiation received. However, the dose absorbed by the body is affected by a number of factors which are used to enhance the efficacy and accuracy of radiation. The quality of radiation (type and energy) directly affects the skin reaction and is related to the amount of energy absorbed by the skin.

The measurement normally used to treat breast cancer is 6 MV photons, which usually means that the maximum radiation dose is being delivered approximately 1.5 cm below the skin surface. Radiotherapy can cause the increase of skin reactions connected to the biological damage done to the epidermis and dermis during the treatment. Normal skin cell repopulation occurs when superficial epithelial cells are shed and replaced by new cells. Erythema can be occur as a result of an acute response to radiation. In the dermis the capillary vessels are dilated and a histamine-like substance is released, which leaves the skin itchy, inflamed, red and warm to the touch (radiation dermatitis). This erythema will become visible within 2–3 weeks of starting treatment. As the cumulative dose of radiotherapy increases, the erythema can change color from a faint to a more pronounced red. After 3–4 weeks of treatments, the radiation will result in further cellular damage, which will appear as dry desquamation. The new cells divide and migrate to the skin surface faster than the old ones are shed, in an attempt to compensate for the skin damage [42,43].

Single cells that remain like the basal layer of the skin after irradiation, multiply rapidly and lead to tissue renewal within 1–2 weeks after treatment. Early complications heal quickly and usually have no further consequences, although they cause serious discomfort to the patient. Late complications become clinically apparent months or even years after irradiation. Redness on the skin may be the result of an individual response to the received dose. All the aforementioned processes related to irradiation and the differences in doses accepted by tissues as well as to the metabolism changes due to started repair processes lead to changes in body temperature.

## 4. Conclusions

The thermal maps obtained during the examination showed the correlation between the dose obtained from the treatment plan and the increased temperature of the treated area. The biggest average temperature increase has been obtained for planned target volume in the third week of the treatment, and it was nearly 0.8 °C.

Apart from the analyzed areas (except isodoses), no increase in temperature was observed in healthy tissues.

The obtained results seem to confirm that the use of thermal imaging in breast cancer radiotherapy treatment may provide some additional information on patients’ response to treatments such as temperature distribution changes which indirectly describe the dynamics of metabolism changes. On the other hand, such information may lead to conclusions about the rebuilding of irradiated tissue, the neoangiogenesis process and the occurrence of an inflammatory state and its range changing. All of these processes may provide information about the healing process and its dynamics.

The study seems to confirm that thermal imaging is useful for evaluating irradiation treatment effects.

## Figures and Tables

**Table 1:** The average temperature changes during radiotherapy in patients in the Planned Target Volume (PTV) area and control group.

	1 Week	2 Weeks	3 Weeks	4 Weeks	Control
patient 1	33.8	33.8	34.5	34.2	32.1
patient 2	33.5	34.5	34.5	34.7	32.0
patient 3	34.4	34.0	35.4	34.7	33.1
patient 4	34.6	34.9	35.3	34.4	33.5
patient 5	34.0	33.4	34.7	34.4	32.7
patient 6	31.9	31.8	32.7	31.8	31.2
patient 7	34.5	34.5	35.2	34.4	32.8
patient 8	34.4	35.2	35.0	34.3	33.6

**Table 2:** Mean temperature increase in the PTV area obtained for the examined patients for each of the isodoses.

PTV Area	40 Gy	30 Gy	20 Gy	10 Gy
0.78 *	0.68 *	0.61	0.64	0.53

* statistically significant at *p* < 0.05.

## Data Availability

Not applicable.
